# Using natural language processing to construct a metastatic breast cancer cohort from linked cancer registry and electronic medical records data

**DOI:** 10.1093/jamiaopen/ooz040

**Published:** 2019-09-18

**Authors:** Albee Y Ling, Allison W Kurian, Jennifer L Caswell-Jin, George W Sledge, Nigam H Shah, Suzanne R Tamang

**Affiliations:** 1 Biomedical Informatics Training Program, Stanford University, Stanford, CA; 2 Department of Biomedical Data Science, Stanford University, Stanford, CA; 3 Department of Medicine, Stanford University School of Medicine, Stanford, CA; 4 Department of Health Research and Policy, Stanford University School of Medicine, Stanford, CA; 5 Center for Biomedical Informatics Research, Stanford University, CA; 6 Center for Population Health Sciences, Stanford University, CA

**Keywords:** cancer distant recurrence, natural language processing, semi-supervised machine learning, electronic medical records, SEER

## Abstract

**Objectives:**

Most population-based cancer databases lack information on metastatic recurrence. Electronic medical records (EMR) and cancer registries contain complementary information on cancer diagnosis, treatment and outcome, yet are rarely used synergistically. To construct a cohort of metastatic breast cancer (MBC) patients, we applied natural language processing techniques within a semisupervised machine learning framework to linked EMR-California Cancer Registry (CCR) data.

**Materials and Methods:**

We studied all female patients treated at Stanford Health Care with an incident breast cancer diagnosis from 2000 to 2014. Our database consisted of structured fields and unstructured free-text clinical notes from EMR, linked to CCR, a component of the Surveillance, Epidemiology and End Results Program (SEER). We identified *de novo* MBC patients from CCR and extracted information on distant recurrences from patient notes in EMR. Furthermore, we trained a regularized logistic regression model for recurrent MBC classification and evaluated its performance on a gold standard set of 146 patients.

**Results:**

There were 11 459 breast cancer patients in total and the median follow-up time was 96.3 months. We identified 1886 MBC patients, 512 (27.1%) of whom were *de novo* MBC patients and 1374 (72.9%) were recurrent MBC patients. Our final MBC classifier achieved an area under the receiver operating characteristic curve (AUC) of 0.917, with sensitivity 0.861, specificity 0.878, and accuracy 0.870.

**Discussion and Conclusion:**

To enable population-based research on MBC, we developed a framework for retrospective case detection combining EMR and CCR data. Our classifier achieved good AUC, sensitivity, and specificity without expert-labeled examples.

## BACKGROUND AND SIGNFICANCE

More than 3.5 million Americans are living with breast cancer, of whom 41 070 (40 610 women and 460 men) died from the disease in 2017.^1^ Breast cancer deaths occur as a result of metastatic breast cancer (MBC)—when breast cancer cells spread to other parts of the body, mostly liver, brain, bones, or lungs.^1^ Patients who were diagnosed with earlier stage breast cancer (stages 0–III) may develop MBC as a distant recurrence of the primary tumor after incident breast cancer diagnosis. Patients who were diagnosed as stage IV breast cancer have *de novo* MBC, as their breast cancers have spread to other parts of the body at the time of initial diagnosis. Despite substantial improvements in the treatment and prognosis of early stage breast cancer, less is known about changes in survival and other outcomes of MBC patients.^2^^,^^3^ Although many clinical trials focus on the treatment of MBC, it is not clear to what extent trial outcomes correspond to real-world outcomes.^4^ In addition, only a small fraction of MBC patients are trial-eligible and it is impossible to study all permutations of sequential drugs in clinical trials. Thus, there is a need to use population-level observational data to study MBC outcomes in this critical patient population.

However, there is a lack of suitable population-based data resources for the study of distant recurrence among breast cancer patients in the United States, as recurrences are not reported in most cancer registries. Although registries from the national Surveillance, Epidemiology and End Results Program (SEER) of National Cancer Institute record the initial cancer stage at diagnosis, the first course of treatment, and up to 1 year follow-up after initial diagnosis, they do not report longer term follow-up, during which time metastatic recurrences would be likely to occur. As a result, population-based cancer registries such as the California Cancer Registry (CCR) of SEER can only be used to identify *de novo* MBC patients, who have been estimated to represent only around one-quarter of all MBC patients^5^^,^^6^ and whose disease may behave very differently from recurrent MBC.^7^ Electronic medical records (EMR) contain large amounts of data collected during routine medical care delivery and have the potential to generate practice-based evidence. However, it has been challenging to make use of this abundance of data in part because of difficulties in identifying which breast cancer patients have had metastatic recurrences.^8^ Thus, the profound gap in our knowledge about real-world treatment of MBC and how patients die of this disease still remains today.

Since identifying MBC cohorts via manual case review is prohibitively laborious, there have been many informatics approaches proposed to retrospectively identify MBC cases from healthcare databases such as claims and EMR. Rule-based approaches that use structured data such as qualifying diagnoses, procedures, and drug codes have been developed.^9–13^ While such approaches are simple to replicate in a new dataset, their reliability is challenged by coding bias and differential coding practices. In addition, these approaches can suffer from low sensitivity (40%–60%), despite reasonable specificity (70%–90%).^10^^,^^11^^,^^13^ A promising alternative is to analyze unstructured clinical text in EMR, which has shown higher sensitivity and specificity.^14^^,^^15^ However, the limitations of this approach include a high cost of initial development, difficulty in adapting to new systems, and most significantly, the requirement for a prohibitively large amount of manually annotated training data.

## OBJECTIVES

We sought to improve upon current informatics approaches to automate MBC case detection with the potential to support population-level surveillance research across California and nationally. To do so, we leveraged the complementary patient data contained in EMR and CCR, and developed a semisupervised machine learning framework, within which we applied natural language processing (NLP) techniques to extract information from unstructured clinical notes. Semisupervised machine learning comprises a class of techniques that make use of unlabeled data to train machine learning models. It falls between unsupervised learning (no labeled training data) and supervised learning (completely labeled training data). It typically consists of pairing a small amount of labeled data with a large amount of unlabeled data. Specifically, our methodological innovation extends the *distant supervision* paradigm described by Mintz et al, which has been applied over a decade in the development of general domain NLP and information extraction tools.^16–18^ In *distant supervision*, a distinct data source can be used to label training examples automatically in the absence of human-labeled training data, for the purpose of subsequent supervised learning.^16–18^ In this study, we implemented *distant supervision* to the problem of retrospective MBC case detection.

## MATERIALS AND METHODS

### Data source

The *Oncoshare* breast cancer research database comprises a three-way data linkage at the patient level. It is an integration of EMRs of Stanford Health Care (SHC), an academic health institution, and multiple sites of the Palo Alto Medical Foundation (PAMF), a community-based medical center in Northern California, both linked to data from CCR, a state-wide SEER registry.^19^^,^^20^ Only SHC patients were included in this study since clinical notes of PAMF patients were not accessible at the time of study. Human Subjects approval for all research reported here was obtained from the Institutional Review Boards of Stanford University and the State of California.

The structured EMR fields in Oncoshare’s clinical database include each patient’s diagnoses, procedures, and drug orders. The unstructured EMR fields include free-text clinician notes such as medical and social histories, impressions, and visit summaries. The CCR contains detailed sociodemographic information such as patient age, race/ethnicity, zip code and neighborhood characteristics, insurance and marital status, tumor characteristics at initial breast cancer diagnosis, and continually updated survival data which SEER obtains through linkage to the Social Security Death Master file and other national databases.^19^^,^^20^

**Figure 1.**
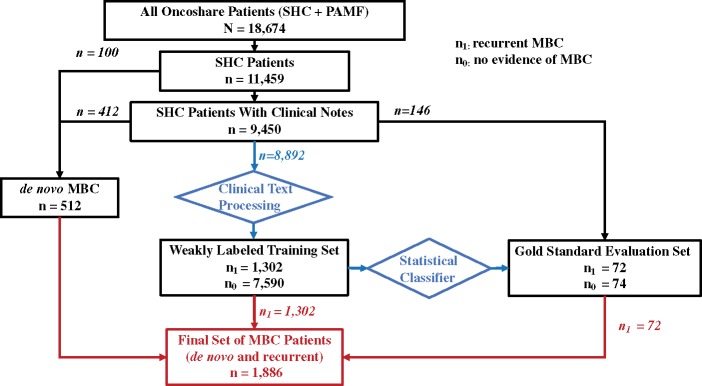
Flowchart of Oncoshare Patient Count by Step. SHC: Stanford Health Care; PAMF: Palo Alto Medical Foundation; MBC; metastatic breast cancer.

For this study, we focused on 11 459 breast cancer patients treated at SHC from 2000 to 2014. Descriptive information on the length of follow-up of the study population appears in Supplementary Tables S1 and S2. Survival status was collected by the CCR as of December 31, 2014 or any later follow-up of specific patients. The last follow-up date was the latest date of the last follow-up from the CCR (December 31, 2014) or from the last encounter date in SHC's EMR database. A flow chart that shows how patients were analyzed by our framework appears in Figure 1. Metastatic disease that was *de novo* stage IV was directly retrieved from the CCR. Patients not identified as *de novo* MBC by CCR and did not have any clinical notes were classified as non-MBC patients. Our informatics method focused on detecting cases of metastatic recurrence, and thus included only patients initially diagnosed in stages 0–III as recorded by the CCR.

### Creating an expert-reviewed “gold standard” patient set for evaluation

Two board-certified medical oncologists (AWK and JLC) manually reviewed deidentified EMRs from 146 female breast cancer patients to create an evaluation set: these patients’ records were not used in the development of the statistical classifiers. The size of this evaluation set was chosen in reference to the validation process of EMR-based phenotypes from the EMRs and Genomics (eMERGE) studies, where 50–200 subjects were reviewed to evaluate the performance of a given algorithm.^21^ The set of 146 patients was a combination of convenient sampling and selective sampling. Approximately 50 MBC cases were selected upon reflection of the participating oncologists’ clinical experience. To extend the size of our evaluation set, we randomly sampled more patients from two ends of a ranked list. Patients with any MBC mentions (positive or negative) were ranked according to the number of positive MBC mentions in their clinical notes. Details on the detection of MBC mentions from clinical notes can be found in the following sections. Considering availability and time constraints of our oncologists, and without prior knowledge of the underlying prevalence of MBC in our study population, we selected patients to achieve a final balanced evaluation set with similar numbers of MBC cases and controls.

The oncologists determined the presence or absence of a metastatic recurrence in each patient’s medical record using all her clinical notes, radiology reports, and pathology reports in the EMR. Unlike note-level or sentence-level review, the participating oncologists synthesized information contained in a patient’s entire medical record available over time before labeling her as having MBC or not. The most common source of information on recurrence was the most recent medical oncology or radiation oncology visit note. If there was no such note, or if this note was written more than 6 months before the time of chart review or the patient's death and did not indicate MBC, then more recent notes from other clinical specialties, pathology reports and imaging reports were examined. If no evidence of MBC was found after review of all these sources, then the patient was labeled as not having recurrent MBC.

### Distant supervision of MBC classification

Our *distant supervision* framework exploits the Oncoshare EMR-CCR linkage. In the absence of a large number of manually annotated cases, we used one data source from the linked data, EMR, to infer a class label for metastatic recurrence. These class labels were then used to supervise the learning of a classification model using input variables from both EMR and CCR.

#### Step 1: Processing EMR clinical notes and assigning distant labels

In step 1, we used NLP-derived features to label patients that were likely to have experienced a metastatic recurrence, based on free-text patient notes in the EMR. Specifically, we adapted an open-source clinical text analysis tool, CLEVER (CL-inical EV-ent R-ecognizer), which has been validated for EMR-based information extraction tasks in prior work, to extract metastatic disease information.^22^ This decision was based on the efficiency of CLEVER’s tagger, which facilitates the review of intermediate system output by subject matter experts and their inclusion in the development of custom clinical NLP extractors. CLEVER’s source code, base terminology, and all customized components that were developed as part of this work are distributed publicly with a MIT software license on Github^1^.

Although mature clinical NLP systems exist, they can be difficult to install and must be adapted to new sources of data. Simple taggers leveraging resources such as the National Library of Medicine’s Unified Medical Language System (UMLS) and SPECIALIST Lexicon tools have been shown to rival their performance and are easier to install.^23^ As illustrated in Supplementary Figure S4, CLEVER makes one modification to these types of general UMLS based taggers such as Noble Coder or MetaMap^24^^,^^25^ in that we pretrained word- and phrase-embedding models on clinical text to expand terminologies that are “seeded” by UMLS terms. Using language-embedding models to identify new terms that were statistically similar to the high quality UMLS seed terms, we developed an enhanced terminology using an iterative and incremental process that included two informaticists and a subject matter expert to assist in the review of candidate terms.

After our terminology for MBC information extraction was complete, we used CLEVER to annotate the corpus and extract mentions of different metastatic disease concepts that could be used to infer the presence or absence of a metastatic recurrence. We also examined their immediate contexts to determine if the target term was negated, hypothetical or an attribute of a family member and not the patient (Supplementary Figure S5). Specifically, CLEVER’s base classes include negation and familial terms from ConText and NegEx that have been expanded through word embedding method to detect additional similar terms directly from patient notes.^22^ The custom classes that we developed for metastatic recurrence detection are shown in Table 1. The CLEVER rule that we developed to assign a case label to each patient was based on the positive present mention of at least one terms from any of the four custom word classes: “METSBONE,” “METSBRAIN,” “METSLIVER,” and “METSLUNG”. These four word classes were constructed in a data-driven way from the most common sites of metastasis among our patients. In contrast to less specific word classes such as “DRECUR,” which contains terms that indicate a nonspecific distant recurrence, these four word classes include terms that indicate both metastatic disease and a location distant to the breast.

**Table 1.** Recurrent metastatic breast cancer term to concept mappings

Custom word class	Short description	Example terms
DRECUR	Distant recurrence	Recurrent metastatic tnbc, distant relapse, distant recurrences, distant metastatic disease involving
LRECUR	Local or regional recurrence	Regional recurrence, nodal recurrence, loco-regional failure, locally recur, in-breast recurrence, local recur
MBC	Metastatic breast cancer	Widespread metastatic breast cancer, widely metastatic triple, metastatic breast carcinoma, metastatic tnbc
MBCLOW	Metastatic breast cancer (low confidence)	Metastic^a^, metasteses^a^, metastatis^a^, metastatic lobular carcinoma, metastatic lesion, metastatic lesions, metastatic foci, mbc, met brca
METSBONE	Metastatic disease to the bone	Bone mets, bone metastasis, bone metasteses^a^, mets to spine, boney mets, diffuse skeletal mets, mets to spine
METSBRAIN	Metastatic disease to the brain	Metastatic disease involving the brain, brain mets, mets to brain, brain metastasis, brain metastases
METSLIVER	Metastatic disease to the liver	Liver mets, liver metastasis, liver metastases, hepatic mets, hepatic metastasis, hepatic metastases
METSLUNG	Metastatic disease to the lung	Mets to lung, pulm mets, lung mets, mbc pulm, lung metastases
METSNOS	Metastatic disease (distant organ not specified)	Widespread metastatic disease, stage4, newly diagnosed metastatic, stage iv
RECUR	Recurrence	Recur, rapid recurrence, multiple recurrences, recurrent disease, reoccurrence, reoccurring
DIED	Death	Passed away, expired on, deceased

a These were original spellings from the clinical notes and the misspellings are left intentionally.

#### Step 2: Recurrent MBC classification

In step 2, we used the distant labels from step 1 to train metastatic recurrence classification models. Two sets of features were included into the classifiers: NLP-derived features from clinical notes in EMR and CCR features. The 427 patient-level NLP-derived features included the total number of terms mentioned in each of the customized word classes (except METSBONE, METSBRAIN, METSLIVER, and METSLUNG) and their frequency as positive or negative concepts, in each specific note type and across all note types in the EMR. The features from the four custom word classes mentioned above were excluded from this step because they were used to infer the training labels, and including them would result in “learning back” our labeling process. The CCR features included structured fields such as age, race, ethnicity, marital status, socioeconomic status, insurance type, comorbidity, year of initial breast cancer diagnosis, cancer stage, tumor grade, tumor histology, and tumor receptor status (eg, expression of estrogen receptor, progesterone receptor, and human epidermal growth factor receptor 2 [HER2/neu]). Missing data in any of the structured features above were coded as a separate category. We trained three classifiers: A (CCR features only), B (NLP features only), and C (NLP + CCR features). Other than having different sets of input features, all aspects of the three classifiers were kept the same for fair comparisons.

We trained logistic regression models with L2 regularization using glmnet package in R.^26^ Compared to regular logistic regression, L2 regularization smoothly shrinks regression coefficients based on regularization parameter, lambda, while retaining all input features in the model.^27^ Such regularization can help reduce prediction error in our case because many of our input features are likely to be correlated. In practice, we chose the largest value of lambda such that error is within 1 standard error of the minimum mean cross-validation error (lambda.1se) by 10-fold cross-validation using the cv.glmnet function. The probability cutoff of the classifier was chosen to optimize the F1 score. Finally, we tested our classifiers on a physician-labeled set of 146 patients (72 cases and 74 controls) and measured model performance using sensitivity, specificity, positive predictive value (PPV or precision), negative predictive value (NPV), and overall accuracy. Bootstrap confidence intervals were calculated for each of the performance measurements by resampling the gold standard sets of patients with replacement 1000 times and taking the 2.5% and 97.5% percentile of the measurements calculated using the bootstrap samples. To benchmark the performance of our classifiers, we also implemented a simple rule-based algorithm to classify MBC patients as those who have at least one instances of 196.XX-199.XX in their structured EMR diagnosis.^12^^,^^28^^,^^29^

## RESULTS

Among the 11 459 patients, follow-up time ranged from 6.3 to 202.8 months with a median of 96.3 months. The mean follow-up time was 97.8 months with a standard deviation 46.7 months. A total of 1, 886 (16.5%) were classified as MBC patients and 9, 573 (83.5%) as not having any evidence of distant metastases in the data that were available to us at the time of this study. Of the 1886 MBC patients, 512 (27.1%) were *de novo* stage IV MBC patients, while 1374 (72.9%) were classified as recurrent MBC patients (1302 from text processing step and 72 reviewed by physicians). This result is consistent with a recent report from the SEER registry using unrelated methods.^6^Table 2 summarizes socio-demographic, clinical and genetic features of patients grouped into MBC (stage 0–III at diagnosis), stage IV at diagnosis, and non-MBC. Using the test set of 146 manually annotated patients, our text processing step generated 15 false-positive and 8 false-negative labels, with an overall accuracy of 0.842 as shown in Table 3.

**Table 2.** Metastatic breast cancer (MBC) case detection results by metastatic breast cancer status

	Recurrent MBC (stage 0–III at diagnosis)		*de novo* MBC (stage IV at diagnosis)		Non-MBC	
	No.	%	No.	%	No.	%
Total	1302	100	495	100	7590	100
Age at diagnosis: mean (SD)	52.99 (13.04)		54.61 (13.62)		55.36 (12.99)	
Year of breast cancer diagnosis						
Before 2005	526	40.40	116	23.43	1738	22.90
2005–2009	463	35.56	189	38.18	2787	36.72
2010–2015	313	24.04	190	38.38	3065	40.38
Race						
White	1025	78.73	394	79.60	5887	77.56
Black	51	3.92	25	5.05	229	3.02
Asian/Pacific Islander	206	15.82	70	14.14	1371	18.06
Other	4	0.31	2	0.40	57	0.75
Missing	16	1.23	4	0.81	46	0.61
Ethnicity						
Hispanic	129	9.91	55	11.11	588	7.75
Non-Hispanic	1170	89.86	439	88.69	6968	91.81
Missing	3	0.23	1	0.20	34	0.45
Neighborhood socioeconomic status^a^						
Lowest quintile	45	3.46	40	8.08	342	4.51
Second quintile	121	9.29	64	12.93	631	8.31
Third quintile	215	16.51	81	16.36	988	13.02
Fourth quintile	254	19.51	105	21.21	1433	18.88
Highest quintile	646	49.62	195	39.39	4015	52.90
Missing	21	1.61	10	2.02	181	2.39
Stage						
0	72	5.53	0	0.00	1813	23.87
I	302	23.20	0	0.00	2837	37.37
II	585	44.93	0	0.00	2186	28.80
III	307	23.58	0	0.00	616	8.10
IV	0	0.00	495	100.00	0	0.00
Missing	36	2.76	0	0.00	141	1.86
Tumor receptor subtype^b^						
Estrogen receptor and/or progesterone receptor (PR)-positive and HER2-negative	608	46.70	247	49.90	3514	46.30
HER2-positive	259	19.89	117	23.64	969	12.77
Triple-negative	223	17.13	63	12.73	689	9.08
Missing	212	16.28	68	13.74	2418	31.86
Grade						
1	163	12.52	21	4.24	1511	19.91
2	446	34.26	167	33.74	3020	39.79
3	580	44.55	177	35.76	2400	31.62
Missing	113	8.68	130	26.26	659	8.68
Histology						
Ductal	1146	88.02	413	83.43	6433	84.76
Lobular	117	8.99	56	11.31	703	9.26
** **Other	39	3.00	26	5.25	454	5.98
Marital status						
Single	202	15.52	95	19.19	1133	14.93
Married	865	66.44	289	58.38	5096	67.14
Divorced	120	9.22	47	9.49	602	7.93
Widowed	81	6.22	44	8.89	518	6.82
Separated, unmarried, or domestic partner	20	1.54	15	3.03	170	2.24
Missing	14	1.07	5	1.01	71	0.94
Payer						
Not insured	11	0.84	3	0.61	35	0.46
Insurance, not otherwise specified	144	11.06	45	9.09	729	9.60
Managed care/HMO/PPO	695	53.38	241	48.69	4489	59.14
Medicaid	121	9.29	60	12.12	390	5.14
Medicare	267	20.51	118	23.84	1610	21.21
Others	21	1.61	11	2.22	115	1.52
Missing	43	3.30	17	3.43	222	2.92

a Neighborhood socioeconomic status (SES) quintile was assigned based on a previously developed measurement by Yost et al for cases diagnosed from 2000 to 2005, and Shariff-Marco et al for cases diagnosed 2006 to 2015.^30^^,^^31^

b Triple negative: estrogen receptor, progesterone receptor and HER2 all negative. HER2 positive: HER2 positive, regardless of estrogen receptor or progesterone receptor status.

**Table 3.** Performance of distant labels and classification models using 146 manually reviewed gold standard patients

		Performance measurements^a^ (95% confidence interval)						
		Area under the curve (AUC)	Sensitivity	Specificity	PPV	NPV	F-1 score	Accuracy
Distant labels		NA	0.889	0.797	0.810	0.881	0.848	0.842
			(0.818, 0.957)	(0.700, 0.889)	(0.723, 0.899)	(0.797, 0.952)	(0.783, 0.908)	(0.781, 0.904)
Classifier	A (CCR)	0.789	0.542	0.824	0.750	0.649	0.629	0.685
		(0.716, 0.861)	(0.423, 0.662)	(0.727, 0.899)	(0.633, 0.863)	(0.543, 0.744)	(0.521, 0.726)	(0.603, 0.760)
	B (NLP)	0.917	0.861	0.878	0.873	0.867	0.867	0.870
		(0.868, 0.966)	(0.778, 0.933)	(0.800, 0.944)	(0.794, 0.943)	(0.783, 0.936)	(0.800, 0.925)	(0.815, 0.925)
	C (NLP + CCR)	0.925 (0.880, 0.969)	0.861	0.878	0.873	0.867	0.867	0.870
			(0.778, 0.933)	(0.800, 0.944)	(0.794, 0.943)	(0.783, 0.936)	(0.800, 0.925)	(0.815, 0.925)

a Note that positive predictive value (PPV), negative predictive value (NPV), F-1 score, and overall accuracy are highly dependent on the prevalence of the condition, which in our case is 72/146 = 0.49. The actual prevalence of recurrent metastatic breast cancer in our study population is likely to be much lower. However, sensitivity, specificity, and area under the curve (AUC) are intrinsic properties of classifier and are insensitive to prevalence of cases.^32^^,^^33^

**Figure 2.**
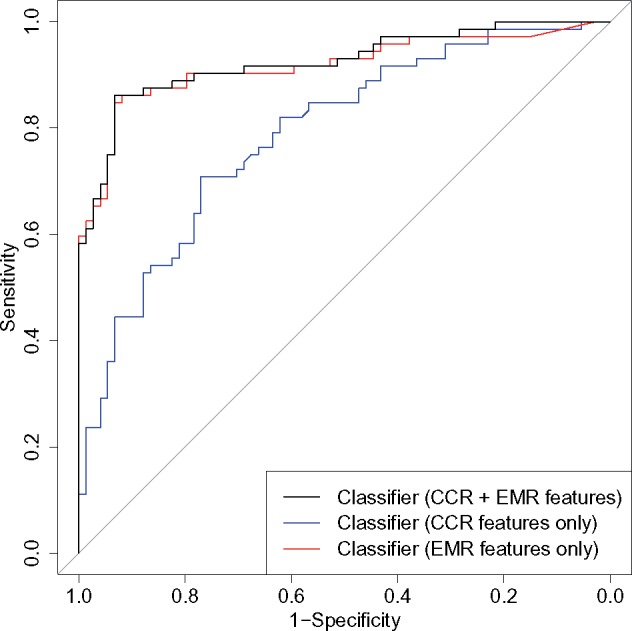
Receiver Operating Characteristic Curve (ROC) of Statistical Classifiers Evaluated using the Test Set of 146 Patients. The area under the curve (AUC) of classifier with CCR and NLP features is 0.925 with 95% confidence interval 0.880–0.969. The AUC of the classifier with CCR features only is 0.789 with 95% confidence interval 0.716–0.861. The AUC of classifier with NLP features is 0.917 with 95% confidence interval 0.868–0.966.

Furthermore, we trained three *distant supervised* classification models for metastatic recurrences using these distantly labeled patients (1302 as MBC and 7590 not enough evidence of MBC) using combinations of CCR and NLP-derived features. A summary of all CCR features used in our classifiers is listed in Table 4. Compared to the classifier A (CCR features only), we observed a boost in all performance measurements by including NLP-derived features in classifiers B and C (Table 3). Classifiers B and C achieved very similar performance regardless of the presence of any CCR features (Table 3). For both classifiers B and C, the regularization parameter lambda was chosen to be 0.041. Using 10-fold cross validation within the training data, we obtained the highest F-1 score of 0.89 with a probability cutoff of 0.45. This cut-off was applied to be evaluated using the 146 manually annotated records in the gold-standard set. Classifiers B and C achieved areas under the receiver operating characteristic curve (AUC) of 0.917 and 0.925 (DeLong 95% confidence interval 0.868–0.966 and 0.880–0.969), respectively (Figure 2).^35^ For both classifiers, there were 9 false-positives and 10 false-negatives, corresponding to sensitivity = 0.861, specificity = 0.878, and overall accuracy = 0.870 (Table 3). The NLP-derived features with the highest beta coefficients from classifier B are shown in Supplementary Table S3. As a benchmark, the ICD-9 code rule-based classifier achieved high sensitivity (0.93) but low specificity (0.47) and PPV (0.63).

**Table 4.** Characteristics of all studied breast cancer patients (*N* = 11 459) derived from the California Cancer Registry^34^

	Stage at diagnosis											
	Stage 0		Stage I		Stage II		Stage III		Stage IV		Missing	
	No.	%	No.	%	No.	%	No.	%	No.	%	No.	%
Total	2335	100	3820	100	3443	100	1120	100	495	100	246	100
Age at diagnosis: mean (SD)	55.31 (11.93)		56.68 (12.97)		53.26 (13.2)		51.77 (12.74)		54.61 (13.62)		56.71 (15.91)	
Year of breast cancer diagnosis												
Before 2005	586	25.10	1106	28.95	1137	33.02	245	21.88	116	23.43	75	30.49
2005–2009	1039	44.50	1396	36.54	1277	37.09	474	42.32	189	38.18	118	47.97
2010–2015	710	30.41	1318	34.50	1029	29.89	401	35.80	190	38.38	53	21.54
Race												
White	1762	75.46	3075	80.50	2699	78.39	871	77.77	394	79.60	191	77.64
Black	66	2.83	109	2.85	114	3.31	53	4.73	25	5.05	10	4.07
Asian/Pacific Islander	472	20.21	599	15.68	586	17.02	179	15.98	70	14.14	32	13.01
Other	21	0.90	23	0.60	17	0.49	5	0.45	2	0.40	11	4.47
Missing	14	0.60	14	0.37	27	0.78	12	1.07	4	0.81	2	0.81
Ethnicity												
Hispanic	161	6.90	256	6.70	327	9.50	124	11.07	55	11.11	29	11.79
Non-Hispanic	2155	92.29	3553	93.01	3104	90.15	995	88.84	439	88.69	206	83.74
Missing	19	0.81	11	0.29	12	0.35	1	0.09	1	0.20	11	4.47
Neighborhood socioeconomic status (SES)^a^												
Lowest quintile	104	4.45	158	4.14	151	4.39	62	5.54	40	8.08	25	10.16
Second quintile	188	8.05	298	7.80	313	9.09	122	10.89	64	12.93	29	11.79
Third quintile	315	13.49	489	12.80	520	15.10	188	16.79	81	16.36	35	14.23
Fourth quintile	468	20.04	735	19.24	666	19.34	230	20.54	105	21.21	46	18.70
Highest quintile	1202	51.48	2062	53.98	1737	50.45	496	44.29	195	39.39	106	43.09
Missing	58	2.48	78	2.04	56	1.63	22	1.96	10	2.02	5	2.03
Tumor receptor subtype^b^												
Estrogen (ER) and/or progesterone receptor (PR)-positive and HER2-negative	118	5.05	2336	61.15	1800	52.28	567	50.63	247	49.90	66	26.83
HER2-positive	48	2.06	545	14.27	651	18.91	254	22.68	117	23.64	22	8.94
Triple-negative	12	0.51	366	9.58	556	16.15	199	17.77	63	12.73	18	7.32
Missing	2157	92.38	573	15.00	436	12.66	100	8.93	68	13.74	140	56.91
Grade												
1	209	8.95	1199	31.39	470	13.65	120	10.71	21	4.24	31	12.60
2	878	37.60	1551	40.60	1356	39.38	387	34.55	167	33.74	50	20.33
3	865	37.04	820	21.47	1420	41.24	521	46.52	177	35.76	64	26.02
Missing	383	16.40	250	6.54	197	5.72	92	8.21	130	26.26	101	41.06
Histology												
Ductal	1989	85.18	3296	86.28	2919	84.78	923	82.41	413	83.43	168	68.29
Lobular	181	7.75	283	7.41	341	9.90	174	15.54	56	11.31	12	4.88
Other	165	7.07	241	6.31	183	5.32	23	2.05	26	5.25	66	26.83
Marital status												
Single	333	14.26	577	15.10	512	14.87	184	16.43	95	19.19	41	16.67
Married	1577	67.54	2555	66.88	2322	67.44	747	66.70	289	58.38	122	49.59
Divorced	184	7.88	301	7.88	293	8.51	100	8.93	47	9.49	27	10.98
Widowed	169	7.24	286	7.49	217	6.30	55	4.91	44	8.89	24	9.76
Separated, unmarried, or Domestic partner	50	2.14	66	1.73	57	1.66	26	2.32	15	3.03	28	11.38
Missing	22	0.94	35	0.92	42	1.22	8	0.71	5	1.01	4	1.63
Payer												
Not insured	11	0.47	17	0.45	22	0.64	6	0.54	3	0.61	5	2.03
Insurance, not otherwise specified	244	10.45	367	9.61	362	10.51	111	9.91	45	9.09	20	8.13
Managed care/HMO/PPO	1455	62.31	2218	58.06	2031	58.99	658	58.75	241	48.69	111	45.12
Medicaid	90	3.85	146	3.82	228	6.62	128	11.43	60	12.12	13	5.28
Medicare	424	18.16	900	23.56	639	18.56	162	14.46	118	23.84	61	24.80
Others	41	1.76	50	1.31	48	1.39	13	1.16	11	2.22	5	2.03
Missing	70	3.00	122	3.19	113	3.28	42	3.75	17	3.43	31	12.60

a Neighborhood socioeconomic status (SES) quintile was assigned based on a previously developed measurement by Yost et al for cases diagnosed from 2000 to 2005, and Shariff-Marco et al for cases diagnosed 2006 to 2015.^30^^,^^31^

b Triple negative: estrogen receptor, progesterone receptor and HER2 all negative. HER2 positive: HER2 positive, regardless of estrogen receptor or progesterone receptor status.

## DISCUSSION

The lack of high-quality longitudinal databases that can be used to study metastatic recurrence is the biggest obstacle to practice-based evidence on how patients die from breast cancer. To address this problem, we developed a novel scalable framework that enables retrospective MBC case detection with good performance (sensitivity = 0.861 and specificity = 0.878). To our knowledge, there has been no one threshold above which the algorithm performance is sufficient for all types of research use. Our framework is flexible in that future researchers could adapt the probability threshold of our algorithm, depending on their needs for sensitivity, specificity, or other performance measures.^11–14^ The contribution of this work is 3-fold. First, we retrieved information from the unstructured text of clinical notes by developing a custom NLP extraction tool for metastatic recurrences and demonstrated the benefit of using unstructured EMR data.^14^ Second, we applied a semisupervised machine learning technique, *distant supervision*, to the problem of metastatic recurrence classification. In doing so, we avoided the salient bottleneck presented by human annotation, which is time and cost-prohibitive for many institutions and researchers. Last, we leveraged complementary data sources, EMR and CCR, to develop a framework for the detection of MBC that enables population-based studies of patients with metastatic cancer.

Given that the two classifiers that included NLP-derived features performed very similarly regardless of the presence of CCR features, we concluded that unstructured data alone was sufficiently informative for the purpose of identifying the recurrent MBC cohort in this study. Note that our primary goal was to solve a binary classification problem (whether a breast cancer patient had experienced metastasis or not) with the best possible performance in order to build a MBC cohort with a diverse set of relevant patient-level variables to be used in subsequent clinical studies. This is in contrast to a classic epidemiologic study, in which researchers quantify the association of common risk factors or discover new ones. Thus, in the classifiers in which CCR features were included, we included all variables from CCR in our penalized logistic regression models that could possibly lead to a better classification model, including known risk factors. Nevertheless, we acknowledge that including known risk factors as predictors in our classifier might cause bias in our cohort construction.

Although additional CCR features only offered marginal gains in the classification performance, we emphasize the importance of taking advantage of both EMR and CCR data resources for the purpose of downstream epidemiological studies. While EMR contains more comprehensive information about patients’ treatment and progression of disease over a longer period of time compared to cancer registry data, the EMR does not reliably collect tumor characteristics.^36^^,^^37^ To be more specific, the main coding system in EMR, ICD, does not specify stage at diagnosis, and metastatic codes do not specify the occurrence of distant recurrence after initial breast cancer diagnosis. Cancer registries such as the CCR are the best data sources to obtain accurate tumor characteristics that are absent in EMR, including breast cancer stage at diagnosis. This information is essential in characterizing any cancer patient cohort and in describing patient outcomes. In addition, the highly accurate and complete sociodemographics information collected by the cancer registry, which is known to be associated with disparities in cancer risk and survivorship, will facilitate downstream outcomes research.^38^ We understand that it can be challenging to link the two datasets due to data privacy issues, but it is possible and Oncoshare is an example that can be replicated at other institutions. We believe such linkage will boost outcomes research in this patient cohort as well as in a broader cancer patient population.^20^^,^^39^^,^^40^

Our work suggests that an important next step is to develop tools for temporal information extraction. Due to the relatively short time between metastatic recurrence and death, NLP approaches must perform at high accuracy to support meaningful survival analysis. Although we initially planned to estimate onset time for metastatic recurrences, we found that simple methods (eg, for a given patient, using the earliest timestamp of all notes with any positive-affirmative MBC mentions) were not sufficient. Analyses of notes from 10 patients found that the most common errors of this naïve approach were attributable to phrases such as “patient was diagnosed with metastatic breast cancer [number] months ago at [another medical institute].” Possible future directions for automating recurrent MBC case detection could be to acquire linguistic annotations of English clinical text or other data for training a temporal metastatic recurrence classification model.

There are several limitations of our study. First and foremost, although we value the external validity of our method, it was prohibitively difficult to conduct any validation studies at the time of this study, due to the lack of similarly linked databases from other institutions as well as restrictions in data sharing. Second, our patients are limited to those treated at SHC, an academic medical center, and thus they do not fully represent the broader community of breast cancer patients from all geographical and socioeconomic backgrounds. Third, even our linked dataset contains incomplete data due to patients receiving care outside of SHC. This is mitigated to some extent by state-wide capture of treatment summaries by CCR, but does not capture events outside of the state. Fourth, our work has primarily focused on NLP-derived features from unstructured free-text data in the EMR and structured data from the CCR. The integration of structured data from the EMR, such as diagnoses, drugs, and procedures that patients received as part of their treatment and continued survivorship care, may also improve classification, especially when there is ambiguity in describing metastasis in the notes or for patients without any clinical notes.^14^ Fifth, we used a relatively simple machine learning classifier: a penalized logistic regression. Use of decision tree analysis and more nuanced machine learning methods may improve classification performance. Sixth, due to practical constraints in selecting our gold standard set, there could be potential bias in evaluating the performance of various algorithms. Last, before we are able to ascertain the date of metastatic recurrence, the performance of our classifier is subject to change depending on the length of follow-up.

## CONCLUSION

In conclusion, we developed an open-source MBC case detection framework using linked EMR-CCR data, within which we used NLP techniques to accurately label breast cancer patients as recurrent metastatic or not. As more linked datasets are developed (eg, the American Society of Clinical Oncology’s CancerLinQ initiative^2^), tools such as ours can readily be adapted for them. This approach has tremendous potential to identify cohorts of recurrent metastatic cancer patients and offer insights into the characteristics, care received, and outcomes of this important and understudied patient population.

## FUNDING

This work was supported by the Breast Cancer Research Foundation; the Suzanne Pride Bryan Fund for Breast Cancer Research; the BRCA Foundation; the Jan Weimer Junior Faculty Chair in Breast Oncology; the Susan and Richard Levy Gift Fund; the Regents of the University of California’s California Breast Cancer Research Program (16OB-0149 and 19IB-0124); and the National Cancer Institute’s Surveillance, Epidemiology and End Results Program under contract HHSN261201000140C awarded to the Cancer Prevention Institute of California. The collection of cancer incidence data used in this study was supported by the California Department of Health Services as part of the statewide cancer reporting program mandated by California Health and Safety Code Section 103885; the National Cancer Institute’s Surveillance, Epidemiology, and End Results Program under contract HHSN261201000140C awarded to the Cancer Prevention Institute of California, contract HHSN261201000035C awarded to the University of Southern California, and contract HHSN261201000034C awarded to the Public Health Institute; and the Centers for Disease Control and Prevention’s National Program of Cancer Registries, under agreement #1U58 DP000807-01 awarded to the Public Health Institute. JLC was supported by an ASCO Young Investigator Award from the Conquer Cancer Foundation and a Damon Runyon Physician-Scientist Training Award. AYL acknowledges Stanford Graduate Fellowship. The ideas and opinions expressed herein are those of the authors, and endorsement by the University or State of California, the California Department of Health Services, the National Cancer Institute, or the Centers for Disease Control and Prevention or their contractors and subcontractors is not intended nor should be inferred.

## CONTRIBUTORSHIP STATEMENT

All authors have met the criteria of authorthip according to International Committee of Medical Journal Editors (ICMJE). Authors AYL. and SRT. have contributed to all stages of the research, including study design, data analysis, results interpretation, drafting, and revision. Authors AWK. and JLC. have made substantial input in the study rationale and conception, data acquisition, and interpretation of the results. Authors GWS and NHS have supervised the study and provided their expertise in breast cancer and informatics respectively. All authors participated in critically reviewing and revising the manuscript, and have approved its final version.

## COMPETING INTERESTS STATEMENT

The authors have no competing interests to declare.

## Supplementary Material

ooz040_Supplementary_Data

## Footnotes

1
https://github.com/stamang/CLEVER

2
https://cancerlinq.org/
