# Supplementary material for: Using natural language processing to construct a metastatic breast cancer cohort from linked cancer registry and electronic medical records data
Source: JAMIA Open. 2019 Sep 18;2(4):528–37. doi: 10.1093/jamiaopen/ooz040 (PMC6994019; doi:10.1093/jamiaopen/ooz040)

# **APPENDIX**

Supplemental Table S1. Length of Follow-Up In Days By Survival Status And Tumor Stage At Initial Diagnosis In Stanford Health Care Patients. Survival status was collected by California Cancer Registry as of 2014-12-31 or any later follow-up of the specific patient. Last follow-up date was the latest date of the last follow-up from California Cancer Registry, 2014-12-31 and last encounter date in Stanford Health Care’ electronic medical record.

|  | | **Stage 0** | **Stage 1** | **Stage 2** | **Stage 3** | **Stage 4** | **Missing** | **Total** |
| --- | --- | --- | --- | --- | --- | --- | --- | --- |
| **Alive** | ***n*** | 2162 | 3351 | 2668 | 695 | 129 | 172 | 9177 |
|  | **min** | 243 | 190 | 282 | 401 | 241 | 393 | 190 |
|  | **max** | 5932 | 6053 | 6083 | 5778 | 5614 | 5921 | 6083 |
|  | **mean** | 2893 | 2847 | 2887 | 2547 | 2200 | 2957 | 2840 |
|  | **sd** | 1343.06 | 1432.91 | 1421.03 | 1268.84 | 1258.82 | 1368.73 | 1397.83 |
| **Dead** | ***n*** | 168 | 468 | 771 | 423 | 360 | 74 | 2264 |
|  | **min** | 562 | 563 | 261 | 459 | 348 | 502 | 261 |
|  | **max** | 5684 | 5748 | 5692 | 5466 | 5468 | 5470 | 5748 |
|  | **mean** | 3530 | 3543 | 3631 | 2928 | 2770 | 3414 | 3330 |
|  | **sd** | 1247.06 | 1289.64 | 1268.87 | 1273.46 | 1391.04 | 1224.44 | 1336.85 |
| **Missing** | ***n*** | 5 | 1 | 4 | 2 | 6 | 0 | 18 |
|  | **min** | 1094 | 4971 | 523 | 464 | 560 | NA | 464.0 |
|  | **max** | 4182 | 4971 | 5222 | 1379 | 3973 | NA | 5222.0 |
|  | **mean** | 2577 | 4971 | 2351 | 921.5 | 1356 | NA | 2069.0 |
|  | **sd** | 1433.99 | NA | 2043.13 | 647.00 | 1308.64 | NA | 1634.97 |
| **Total** | ***n*** | 2335 | 3820 | 3443 | 1120 | 495 | 246 | 11459 |
|  | **min** | 243 | 190 | 261 | 401 | 241 | 393 | 190 |
|  | **max** | 5932 | 6053 | 6083 | 5778 | 5614 | 5921 | 6083 |
|  | **mean** | 2938 | 2933 | 3053 | 2688 | 2604 | 3094 | 2935 |
|  | **sd** | 1346.22 | 1434.45 | 1423.14 | 1284.59 | 1384.07 | 1341.04 | 1400.37 |

Supplemental Table S2. Length Of Follow-Up In Days By Survival Status And Insurance Status In Stanford Health Care Patients

|  | | **Insured*** | **Not Insured** | **Others/Missing** | **Total** |
| --- | --- | --- | --- | --- | --- |
| **Alive** | ***n*** | 8691 | 40 | 446 | 9177 |
|  | **min** | 190 | 641 | 365 | 190 |
|  | **max** | 6083 | 5622 | 5978 | 6083 |
|  | **mean** | 2815 | 2935 | 3301 | 2840 |
|  | **sd** | 1393.37 | 1376.43 | 1409.20 | 1397.83 |
| **Dead** | ***n*** | 2125 | 24 | 115 | 2264 |
|  | **min** | 261 | 1456 | 714 | 261 |
|  | **max** | 5748 | 5403 | 5464 | 5748 |
|  | **mean** | 3313 | 3863 | 3534 | 3330 |
|  | **sd** | 1337.65 | 1136.35 | 1330.85 | 1336.85 |
| **Missing** | ***n*** | 16 | 0 | 2 | 18 |
|  | **min** | 464 | NA | 778 | 464.0 |
|  | **max** | 5222 | NA | 1094 | 5222.0 |
|  | **mean** | 2210 | NA | 936 | 2069.0 |
|  | **sd** | 1683.36 | NA | 223.45 | 1634.97 |
| **Total** | ***n*** | 10832 | 64 | 563 | 11459 |
|  | **min** | 190 | 641 | 365 | 190 |
|  | **max** | 6083 | 5622 | 5978 | 6083 |
|  | **mean** | 2912 | 3283 | 3340 | 2935 |
|  | **sd** | 1397.23 | 1359.90 | 1400.46 | 1400.37 |

*Insured: Insurance, NOS, Managed care/HMO/PPO, Medicaid, and Medicare

Supplemental Table S3. Top Classification Features From Classifier B (only NLP-derived features included)

| **Rank** | **Feature** | **Explanation of Feature** | | | **Regression Coefficient** | **Exponential of Regression Coefficient** |
| --- | --- | --- | --- | --- | --- | --- |
|  |  | **Type of Mentions** | **Custom Word Class** | **Note Type** |  |  |
| 1 | MBCuniPN | Indicator variable that there are more unique positive mentions than negative ones | MBC: metastatic breast cancer | All note types | 3.293 | 26.933 |
| 2 | METSNOSPosCyto | Number of positive mentions | METSNOS: metastatic disease (distant organ not specified) | Cytology notes | 0.946 | 2.575 |
| 3 | MBCPosOutSR | Number of positive mentions | MBC: metastatic breast cancer | Outpatient screen review notes | 0.830 | 2.292 |
| 4 | LRECURPosOutSR | Number of positive mentions | LRECUR: local or regional recurrence | Outpatient screen review notes | 0.734 | 2.083 |
| 5 | LRECURPosMNR | Number of positive mentions | LRECUR: local or regional recurrence | Magnetic nuclear resonance notes | 0.710 | 2.034 |

* For example, the highest ranked feature MBCuniPN is a binary indicator variable that takes on the value 1 if there are more unique positive mentions than negative ones of the terms in the custom word class MBC, metastatic breast cancer (Table 1), across all types of clinical notes. Compared to patients with MBCuniPN = 0, the odds of patients with MBCuniPN = 1 having recurrent metastatic breast cancer is 26.933 times higher, holding all other variables constant in the classification model.

** Since mentions of words from these four word classes “METSBONE”, “METSBRAIN”, “METSLIVER”, and “METSLUNG” were used to determine the weak labels, none of the input features into the classifier used information regarding to features in these four word classes.

*** Compared to logistic regression models without regularization, interpretation of these coefficients needs to be proceeded with caution, as L2 regularization reduces the regression coefficients of correlated variables.

Supplemental Figure S4. Corpus-driven Expansion of Metastatic Breast Cancer Seed Terms Using Word and Phrase Embeddings

#
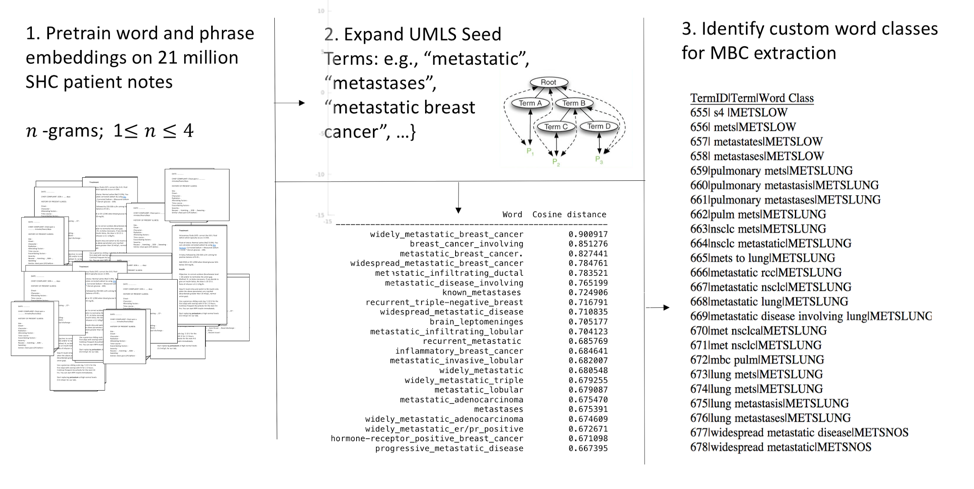


Supplemental Figure S5. An Example Text Snippet Processed by CLEVER to Extract Information on Metastatic Recurrence. An illustration of the application of CLEVER to extract information from clinical text. Given a snippet from a patient’s notes, CLEVER detects target terms (e.g skeletal metastases) in one of the customized word classes (e.g. METSBONE), together with their locations in the clinical note and any modifying words and punctuation around it (e.g. negation). As a result, each clinical note is machine annotated with the presence or absence of mentions of key clinical concepts, considering relevant semantic modifiers in its immediate context.


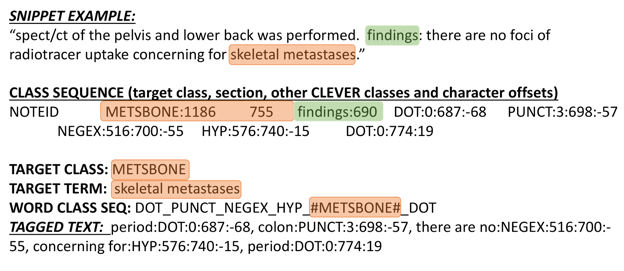

Supplement: ooz040_Supplementary_Data [file ooz040_supplementary_data.docx]
